# Pre-exposure prophylaxis in France: How many MSM are eligible and how much will it cost?

**DOI:** 10.1371/journal.pone.0278016

**Published:** 2022-12-01

**Authors:** Youssoufa M. Ousseine, Nathalie Lydié, Annie Velter

**Affiliations:** Santé Publique France, French National Public Health Agency, Saint-Maurice Cedex, France; URCEco Ile de France Hopital de l’Hotel Dieu, FRANCE

## Abstract

**Background:**

Pre-exposure prophylaxis (PrEP) was definitively authorized in France in 2017 after a two-year probationary period. The fact that the estimated number of MSM eligible for PrEP is still unknown is a barrier to this prevention tool’s roll-out at the national level. This study aimed to estimate the number of MSM eligible for PrEP in France, and to evaluate the direct cost of its roll-out.

**Methods:**

We used data from several sources including the *Enquête Rapport au Sexe 2019-ERAS 2019* survey, the 2019 French population census from National Institute of Statistics and Economic Studies (*INSEE)*, and the National Public Health Agency’s (*Santé Publique France*) 2016 health barometer survey. We also used data from previous studies which estimated the proportion of MSM who were sexually active in the 12 months prior to the studies, and HIV prevalence in MSM in France. Furthermore, we used data on PrEP drug costs from the French public drug database and data on medical examinations costs from the IPERGAY study.

**Results:**

For 2019, the number of HIV seronegative MSM in France who were sexually active in the previous 12 months was estimated at 398,015. Of these, 142,379 (95%CI: 139,893–145,241) and 104,645 (95%IC: 102311–106979) were eligible for PrEP, based on the Menza score and on official French criteria, respectively. The overall estimated cost of PrEP roll-out in eligible MSM varied between € 317,685,216 and € 545,903,216 for official French criteria, which was higher than the estimated €432,240,851 and €742,753,074 according to the Menza score.

**Conclusions:**

Our estimations will enable policy makers to make evidence-based decisions about PrEP roll-out to MSM in France. To accelerate the process, it is important to decentralize PrEP delivery, authorize general practitioners to write prescriptions, and promote this prevention tool through information campaigns.

## Background

Although great progress has been made in recent decades to reduce HIV transmission, incidence is still relatively high in France. An estimated 6,200 cases were diagnosed in 2018, down 7% from 2017 [1]. This decline varied according to region and population category. The fact that incidence was highest in men who have sex with men (MSM) highlights the need to increase tailored HIV prevention policies for this population.

The HIV prevention arsenal has expanded in recent years with the advent of pre-exposure prophylaxis (PrEP), and the recognized benefits of treatment as prevention (TasP) [2–7]. Several countries and health institutions recommend PrEP for high-risk populations [8–12]. In 2015, France became the second country in the world—after the Unites States of America- to offer PrEP. Initially provided on an off-label basis using France’s Temporary Recommendation for Use system, its prescription was fully approved in April 2017 [13, 14]. Given the high HIV incidence in MSM in France, they are considered a priority group for PrEP roll-out and its uptake has grown in this population since 2015 [15].

Initially in France, PrEP initiation was restricted only to specialized hospital practitioners. Since June 1, 2021 authorities opened it to general practitioners in order to accelerate its implementation by providing easier access [16]. This prescription generalization raises a number of questions, in particular the economic cost of providing PrEP to all eligible individuals.

While several studies have estimated the population size of MSM in different countries, few have estimated how many MSM are eligible for PrEP [17, 18]. The European EMIS-2017 survey estimated the difference—for several countries—between the proportion of MSM PrEP users actually using PrEP and the proportion of MSM who declared they would be ‘very likely’ to use PrEP if they could access it [19]. The estimated difference for France was 12%. The authors did however point out several limitations in the methodology used for their estimations.

In order to accompany PrEP roll-out in the French MSM population, it is important to i) identify which MSM are most at risk of HIV infection are therefore are the (most) eligible to start PrEP, as well as their approximate population size; ii) estimate their approximate population size; iii) predict the resulting budgetary impact of rolling out PrEP to all this population.

This study aimed to estimate the population size of MSM eligible for PrEP in France using two different approaches—namely the Menza score [20] and current official French criteria [8]—and to approximate the direct cost of prescribing PrEP to all this population according to each approach.

## Methods

### Data sources and measures

Data and input indicators for this study came from the following sources:

- The *Enquête Rapport au Sexe 2019* (ERAS 2019) survey. ERAS 2019 is a large, cross-sectional online survey of MSM in France conducted in 2019. It aimed to evaluate, over time, the uptake of diversified prevention by MSM in France.
  It was anonymous, self-administered and voluntary. This study followed the ethical guidelines set out in the 1975 Helsinki Declaration. The online survey protocol of the ERAS survey was evaluated and approved by the institutional review board of *Santé Publique France* (Ref: DPPS-09. Enquête Rapport au sexe.). Participants were recruited through private messaging and banners on several social networking and dating websites, as well as dating applications for gay and bisexual men. By clicking on a link or banner, the participant was directed to the survey site, which contained information about the aims and contents of the survey, the terms of participation and data privacy. By clicking on a button containing the text “*I have read and understood the information above*” the participant provided informed consent and was directed to the online questionnaire. No IP addresses were collected. The recruitment campaign lasted six weeks, from February 16 to March 31, 2019. Inclusion criteria were being an MSM and being aged 18 years and over. The following data were collected: i) sociodemographic characteristics, ii) HIV testing history, iii) self-reported HIV status, iv) number of sexual partners in the previous six and 12 months, v) type of male sexual partner(s) (i.e., casual/stable), vi) HIV status of male partner(s), vii) sexual practices (anal intercourse, fisting, other hardcore sex practices) during most recent intercourse, viii) chemsex (i.e., drug consumption while having sex) during most recent intercourse, and finally ix) STI testing and prevention behaviors, including condomless anal intercourse (CAI), PrEP use, TasP, and post-exposure prophylaxis (PEP) use.
- The National Institute of Statistics and Economic Studies (*INSEE*) population census, which provided an estimate for the number of resident males in France, according to different age groups, as of January 1, 2019 [21].
- The National Public Health Agency’s (*Santé Publique France*) 2016 Health Barometer survey, which provided the proportion of MSM in the country and the proportion of MSM sexually active in the previous 12 months among MSM [22]. The Health Barometer is a repeated national survey conducted by telephone among a representative sample of the population aged 15 to 75 years old residing in metropolitan France. It provides an accurate estimate of the proportion of MSM in the country.
- A 2016 study conducted by the ANRS in France, which estimated HIV prevalence in MSM in France [23]. The study was based on statistical modeling using large national data sources, specifically, the national HIV surveillance system database, the general social insurance scheme database, and the French HIV hospital database.
- The public drug database which reports drug costs and was consulted in 2021, and the IPERGAY study which reported medical examination costs for 2016 [24, 25].

### Analyses

#### Step 1: Estimation of number of HIV-seronegative MSM in France sexually active in the previous 12 months

We multiplied the 2016 Health Barometer survey’s estimate for the proportion of MSM sexually active in the previous 12 months [22] by the INSEE’s 2019 estimate for the number of males aged 18–69 years old in the French general population to obtain an estimate for the total population of MSM sexually active in the previous 12 months in France [21]. From this tabure, we subtracted the estimated number of HIV-positive MSM (calculated by applying the 2016 ANRS study’s estimate for HIV prevalence in MSM in France [23]) in order to arrive at an estimate for the number of HIV-seronegative MSM in France who were sexually active in the previous 12 months.

#### Step 2: Estimation of the proportion of MSM who participated in the ERAS 2019 survey that were eligible for PrEP

**Fig 1:**
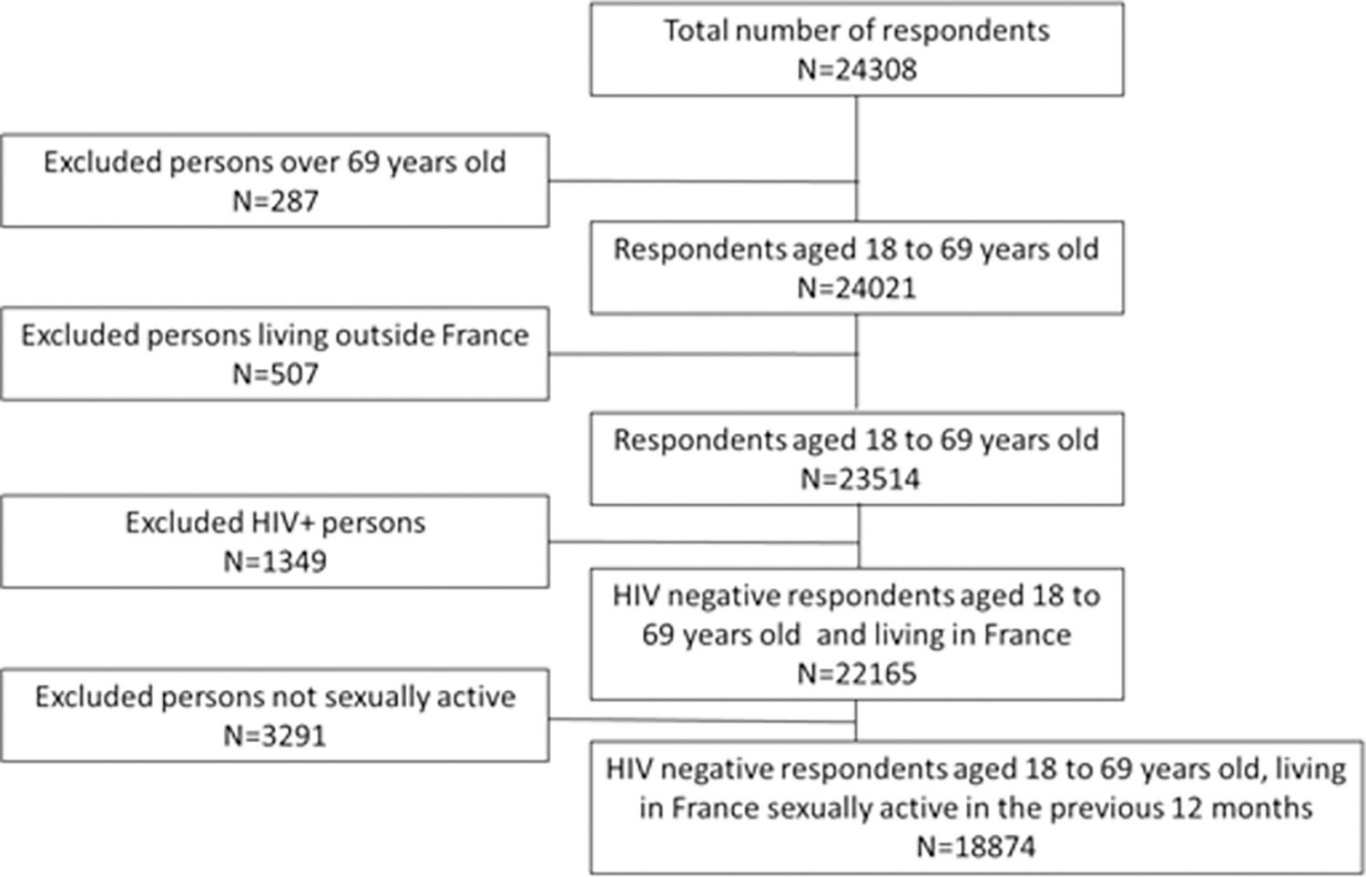
Participant’s flow chart in ERAS 2019 survey.

We estimated the proportion of MSM in the ERAS 2019 survey that were eligible for PrEP (understood here as MSM with the highest risk of HIV infection). Men who were sexually active in the previous 12 months and aged 18 to 69 years old in the ERAS 2019 were the base population for Step 2 (Fig 1).

To estimate this proportion, we used two different approaches. In the first approach, we calculated the proportion of people at greatest risk of acquiring HIV with the Menza score [20]. In the second, we used current official French criteria for PrEP eligibility [8]. The criteria for each method are presented in Table 1.

**Table 1:** Menza score items and official French criteria.

Menza score				Official French criteria	
Menza item	Self-reported indicators in 2019 ERAS which were selected as proxies for Menza items	Response	Points	Official French criteria item	Self-reported indicators in 2019 ERAS which were selected as proxies for official French criteria
Does your patient/client report 10 or more male sexual partners in the prior year?	Reporting 10 or more male sexual partners in the previous 12 months	Yes	3	Using HIV post-exposure treatment (PEP) at least once in previous 12 months	Using HIV PEP during most recent AI
		No	0		
Does your patient/client report unprotected AI with a partner with positive or unknown HIV status in the prior year?	Unprotected AI with a partner with positive or unknown HIV status during most recent AI	Yes	1	CAI with at least two different sexual partners in previous six months	At least one unprotected AI with a casual partner and at least two partners in the previous six months
		No	0		
Has your patient/client used methamphetamine or inhaled nitrites (popper) in the prior 6 months?	Used a psychoactive product (cocaine, GHB/GBL, amphetamines, MDPV, 3-MMC, 4–4-MMC, etc.) during most recent AI	Yes	11	Drug use during sex (Chemsex)	Using a psychoactive product (cocaine, GHB/GBL, amphetamines, MDPV, 3-MMC, 4–4-MMC, etc.) during most recent AI
		No	0		
Does your patient/client have gonorrhea, chlamydia or syphilis, or does he have a history of these infections?	Diagnosis of gonorrhea, chlamydia or syphilis in previous 12 months	Yes	4	STI in previous 12 months	At least 2 STI diagnosed in previous 12 months
		No	0		
**Min-Max score**			0–19		
**Threshold for eligibility**			**>1**	**Meeting at least one of the four criteria**	

AI: anal intercourse; CAI: condomless anal intercourse; STI: sexually transmission infections.

#### Step 3: Estimation of the number of MSM eligible for PrEP in France

We then calculated the number of MSM eligible for PrEP in the whole of France, by multiplying the estimated proportion of MSM eligible for PrEP in ERAS 2019 (the estimate in Step 2 above) by the estimated number of HIV-seronegative MSM in France who were sexually active in the previous 12 months (the estimate in Step 1 above).

#### Step 4: Direct cost estimation

Finally, we estimated the annual direct cost to France’s national health insurance fund if PrEP were provided to all eligible MSM (the estimate in Step 3 above). This was not a budget impact analysis or efficiency analysis, but simply a crude estimation, which took into account the cost of drugs reported in the public drug database for 2021, and the costs of medical examinations reported for 2016 reported in the IPERGAY trial (Training including a pre-PrEP workup about ELISA, HBV, HCV, alanine aminotransferase, and creatinin, information kit—cost 738€; HIV ELISA tests—cost 126€; HIV Plasma viral loads—cost 16€; Diagnosis of STIs (Chlamydia and gonococcal infections, HBV, HCV, syphilis)—cost 262€) [24, 25].

## Results

Overall, 24,308 respondents completed the ERAS 2019 survey. Among them, 23,514 respondents were aged 18 to 69 years and living in France. Of these, 18,874 (78%) participants were HIV seronegative and sexually active in the previous 12 months (Fig 1).

As of January 1, 2019, the number of men in France (INSEE data) between aged between 18 and 69 was estimated at 20,377,923 (Table 2). We calculated that approximately 2.3% (468,692) of them were MSM who had been sexually active in the previous 12 months. HIV prevalence in MSM in France was estimated at 17%. This corresponded to 79,678 HIV-positive MSM who had been sexually active in the previous 12 months.

**Table 2:** Estimation of number of HIV-seronegative MSM in France sexually active in the previous 12 months.

	Source	Proportion	Total number	95% CI
Male population 18–69 years old living in Metropolitan France on January 1, 2019	INSEE 2019 [21]		20,377,923	
Proportion of sexually active MSM in previous 12 months	Bajos et al., 2018 [22]	0.023	468,692*	
HIV prevalence among MSM in France	Supervie et al., 2018 [23]	0.17	79,678*	
Population of HIV negative MSM sexually active in the previous 12 months			**389,015** *	

* Rounded value without decimal point.

We estimated the population of HIV seronegative MSM in France who were sexually active in the previous 12 months in 2019 at 389,015.

The proportions of respondents eligible to initiate PrEP in the ERAS 2019 survey, according to the two different approaches used, are presented in Table 3. For the Menza score approach, 36.6% (95%CI: 36.0–37.3) MSM were eligible for PrEP (score >1), while 26.9% (95%CI: 26.3–27.5) were eligible according to the official French criteria approach. For the latter, the majority of MSM eligible for PrEP (4523) reported CAI with at least two different partners in the previous six months. Sixty-nine (69) MSM reported using PEP.

**Table 3:** Estimation of proportion of MSM eligible for PrEP in ERAS 2019 survey.

** **	** **	**Total number**	**Proportion**	**95% CI**
**Non-HIV positive respondents aged 18 to 69, living in France and sexually active in the previous 12 months (ERAS 2019)**	** **	**18,874**	**100%**	** **
**Official French criteria**				
CAI with at least two different sexual partners in the previous six months		4,523		
At least two diagnosed STI in the previous 12 months		590		
Used a psychoactive product during most recent AI		781		
Used HIV post-exposure treatment (PEP) during most recent AI		69		
**MSM eligible for PrEP**	**Official French criteria (meeting at least one of the four criteria)**	**5079**	**26.9%**	**[26.3–27.5]**
	**Menza risk (score >1)**	**6,917**	**36.6%**	**[36.0–37.3]**

Among HIV seronegative MSM in France who were sexually active in the previous 12 months in 2019, 142,379 (95%CI: 139,893–145,241) (36.6%) and 104,645 (95%IC: 102311–106979) (26.9%) were eligible to initiate PrEP, based on the Menza score and French official criteria approaches, respectively.

Projection costs (for 2021) are presented in Table 4. The monthly price of PrEP per person in France varied between €157.82 and €339.56. The cost of PrEP per person per year varied between €1894 and €4075. Other costs including training of healthcare professionals (pre-PrEP workshop about ELISA, HBV, HCV, alanine aminotransferase, creatinine, and prevention kits (which include information, condoms and lubricant)), HIV ELISA tests, HIV Plasma viral load measurements, and diagnosis of STI (Chlamydia and gonococcal infections, HBV, HCV, syphilis) were estimated at €1142 per person per year. According to the official French criteria estimations, the overall cost varied between € 317,685,216 and € 545,903,216, while the Menza score approach gave a higher overall estimated cost, varying between €432,240,851 and €742,753,074.

**Table 4:** Estimation of annual cost of rolling out PrEP to all eligible MSM in France in 2019.

		Annual cost per person (€)			Global cost (€)	
Available drug in France (*all film-coated tablets*)	Price^a^ [24]	Drug cost	Other costs ^b^ [24]	Annual costs	Official French criteria	Menza Score
TRUVADA Gilead 200 mg/245 mg	339.56	4,075	1,142	5,217	545,903,216	742,753,074
EMTRICITABINE TENOFOVIR DISOPROXIL MYLAN 200 mg/245 mg	168.93	2,027	1,142	3,169	331,636,476	451,222,863
EMTRICITABINE/TENOFOVIR DISOPROXIL BIOGARAN 200 mg/245 mg	168.93	2,027	1,142	3,169	331,636,476	451,222,863
EMTRICITABINE/TENOFOVIR DISOPROXIL EG 200 mg/245 mg	168.93	2,027	1,142	3,169	331,636,476	451,222,863
EMTRICITABINE/TENOFOVIR DISOPROXIL KRKA D.D. 200/245 mg	157.82	1,894	1,142	3,036	317,685,216	432,240,851
EMTRICITABINE/TENOFOVIR DISOPROXIL SANDOZ 200 mg/245 mg	168.93	2,027	1,142	3,169	331,636,476	451,222,863
EMTRICITABINE/TENOFOVIR DISOPROXIL TEVA 200 mg/245 mg	168.93	2,027	1,142	3,169	331,636,476	451,222,863
EMTRICITABINE/TENOFOVIR DISOPROXIL ZENTIVA 200 mg/245 mg	168.93	2,027	1,142	3,169	331,636,476	451,222,863

a: Prices published in the French public drug database, http://base-donnees-publique.medicaments.gouv.fr/afficheGroupeGene.php?idGrp = 1371.

b: Training including a pre-PrEP workup about ELISA, HBV, HCV, alanine aminotransferase, and creatinin, information kit—cost 738€; HIV ELISA tests—cost 126€; HIV Plasma viral loads—cost 16€; Diagnosis of STIs (Chlamydia and gonococcal infections, HBV, HCV, syphilis)—cost 262€.

total cost = 738+126+16+262 = 1142€.

## Discussion

This is the first study in France to estimate the population size of MSM who are eligible to initiate PrEP using two different methods, and to estimate the projected global annual cost to the country’s health insurance system of rolling out PrEP to all this population. According to the Menza score, an estimated 142,379 MSM were eligible to initiate PrEP in 2019, and depending on the drug used, the related global annual cost varied between €432,240,851 and €742,753,074. Instead, based on official French criteria, an estimated 104,645 MSM were eligible to initiate PrEP in 2019, with a global annual cost of between € 317,685,216 and € 545,903,216, again depending on the drug used.

PrEP was definitively authorized in France in 2017 after a probationary period of two years. The most recent available data from the French National Agency for Drug Safety (ANSM) reported that in 2019 the number of PrEP users was six times lower (20 478) than the number of people eligible [26]. This huge gap is mostly the consequence of structural, cultural and stigma-related factors [27]. Although general practitioners could refill prescriptions, only specialist physicians could prescribe PrEP in France, and initiation—whether with Truvada or generic equivalents—mostly occurs in hospitals (in 90% of cases) or in specialized CeGIDD centers (Free Information, Screening and Diagnosis Centers) [28]. Furthermore, there are geographic disparities in access to this prevention tool [29]. However, in order to accelerate its roll-out and increase PrEP users, French health authorities extended its prescription by general practitioners since June 1, 2021 [16].

To anticipate PrEP roll-out and scale-up, it is therefore crucial to estimate the eligible population size. Previous studies estimated that between 32,000 and 50,000 MSM in France were at high risk of HIV infection and needed PrEP [28, 30]. Another study based on people’s intention to take PrEP estimated that 12% of MSM in France were eligible to initiate it [19]. These numbers were most probably biased and underestimated the true figures, because they were all based on a single self-reported criterion (which differed for each study) which was most likely related to the respondents’ perception of their own risk of HIV acquisition and/or their perception of the benefit of taking PrEP [31]. Furthermore, a study conducted in Belgium showed an incongruence in PrEP eligibility data which were based on self-reported risk perception and formal criteria [32]. However, the formal criteria are also self-reported.

To avoid this bias, in the present study—which was based on objective criteria—we chose to use two approaches to estimate the eligible MSM population size in France: the official French criteria, and the validated Menza score, which is used by clinicians to assess PrEP eligibility. We noted a small difference, with the Menza score approach providing slightly higher estimates. This reflects a previous study, where the Menza score approach gave higher estimates than other HIV risk assessment tools [31]. These consistently higher estimates might be related to the construction of our two measurement tools. More specifically, the threshold value of one which is used in the Menza score could be too broad a criterion, and therefore include too many people. Furthermore, the minimum number of STI considered in the STI criterion in the Menza score approach is one, whereas in the official French criteria it is two. In addition, the difference observed between two methods was artificially underestimated because some Menza criteria are lifetime or prior 12/ 6 months, while the data used to attribute the score were during most recent AI. Our estimates are close to those in another French study and in an Australian study, which estimated that 35 and 31% of MSM were eligible to initiate PrEP, respectively [18, 29]. The marginal differences between our estimates and theirs can be explained by the fact that indicators and measurement periods differed between all three studies. This fact may also explain the difference between our estimates and that found in a study on eligible MSM in the USA, which was 25% [33].

Using the official French criteria approach, 89% (4,523 on 5,079) of eligible MSM in our study met the CAI criterion of having at least two different partners in the previous six months. This reflects the abovementioned Australian study, which showed that the majority of MSM eligible to initiate PrEP practiced CAI [18]. However, focusing on only a single criterion (i.e., CAI) would pose a problem, as it would exclude people with other HIV-risk behaviors, and this could comprise prevention policies. The criteria for assessing the population at risk of HIV infection vary from study to study and from country to country. Sometimes using one criterion can exclude people who meet another. Given that these criteria only take into account past risk and not potential future dynamics and changes in prevention behavior, we think that PrEP can be useful for anyone in certain sexual life contexts, taking into account all sexual orientations and with all gender identities [34].

It is sometimes difficult to predict the overall cost of a public health measure at the country level. In the present study, we estimated how many MSM are eligible for PrEP in France and how much rolling out this prevention tool to all this population would cost the country’s social security system. We found that using current official French criteria for PrEP eligibility, roll-out to the estimated 122,929 eligible MSM in the country would cost between € 317,685,216 and € 545,903,216. This variation is due to the variation between PrEP drug prices. Indeed, the price of generic drugs could differ by a factor of two in our study; for example, the monthly price for KRKA DD was €157.82 unlike €339.56 for Gilead. However, this global cost was not definitive, because it did not take into account external costs, including possible STI treatment. Furthermore, it is difficult to anticipate the costs related to organizational changes (improved accessibility, reduced territorial inequalities, etc.) which roll-out would entail [29].

Our results provide policy makers with a preliminary financial projection. Further work is needed to complete this economic projection.

Our study has limitations. First, the data used in the 2016 ANRS study for HIV prevalence in MSM in France were actually collected in 2010 [23], and may not reflect more recent trends. However, a study conducted in Paris in 2013 reported a similar prevalence [35]. This suggests the possibility of a stable—albeit high—prevalence rate over time. Second, some of the indicators used in the ERAS 2019 survey were proxies for the criteria investigated, and were based on self-reported HIV status (see S1 Appendix). Third, the estimation of the HIV-negative MSM population is made by subtracting the number of estimated HIV-infected MSM to the total of sexually active last 12 month MSM, given that not all HIV-infected MSM may have been sexually active in the past 12 months. Fourth, changes in the prices of medical examinations and check-ups might influence our overall cost estimates. However, working with data from the recent national IPERGAY trial should have largely minimized any such variations [24]. Finally, we focused only on MSM in this study. The lack of data for populations of all sexual orientations and gender identities, who are at high risk of acquiring HIV, prevented us from being able to also estimate the population sizes in terms of PrEP eligibility.

## Conclusion

We estimated that between 104,645 and 142,379 MSM in France were eligible to initiate PrEP in 2019, with a global annual cost of between € 317,685,216 and € 742,753,074. Our results should enable policymakers to take evidence-based decisions about PrEP roll-out and scale-up in France. To accelerate roll-out, it is important to decentralize PrEP delivery, authorize its prescription by general practitioners, and promote this prevention tool through information campaigns.

## Supporting information

S1 Appendix Questions in ERAS survey used as proxy. AI: anal intercourse; TasP: treatment as prevention; PrEP: pre-exposure prophylaxis; GHB: gamma-hydroxybutyrate; GBL: Gamma-butyrolactone; MDPV: Methylenedioxypyrovalerone; 3-MMC: 3-methylmethcathinone; 4-MMC: 4-methylmethcathinone; HPV: human papillomavirus.
(DOCX)
