# Supplementary material for: Pre-exposure prophylaxis in France: How many MSM are eligible and how much will it cost?
Source: PLoS One. 2022 Dec 1;17(12):e0278016. doi: 10.1371/journal.pone.0278016 (PMC9714876; doi:10.1371/journal.pone.0278016)
Supplement: S1 Appendix — AI: anal intercourse; TasP: treatment as prevention; PrEP: pre-exposure prophylaxis; GHB: gamma-hydroxybutyrate; GBL: Gamma-butyrolactone; MDPV: Methylenedioxypyrovalerone; 3-MMC: 3-methylmethcathinone; 4-MMC: 4-methylmethcathinone; HPV: human papillomavirus. (DOCX) [file pone.0278016.s001.docx]

**S1 Appendix. Questions in ERAS survey used as proxy**

| **Menza score** | | **Official French criteria** | |
| --- | --- | --- | --- |
| **Menza item** | **Self-reported indicators: Questions in ERAS 2019 survey used** | **Official French criteria item** | **Self-reported indicators : Questions in ERAS 2019 survey used** |
| Does your patient/client report 10 or more male sexual partners in the prior year? | **How many male sexual partners have you had in the last 12 months**?  Response range : 0 – 1000 | Using HIV post-exposure treatment (PEP) at least once in previous 12 months | **Did you use post-exposure treatment after your last AI?**  Response: Yes; No |
| Does your patient/client report unprotected AI with a partner with positive or unknown HIV status in the prior year? | **During you last AI, did you use...?Multiple responses are possible**  Responses:  Condom; TasP; PrEP; None of these means of prevention;  **With respect to HIV, your partner at last AI was…?**  Responses:  HIV-negative (not infected with HIV)  Seropositive (was infected with HIV)  Don't know | CAI with at least two different sexual partners in previous six months | **How many male sexual partners have you had in the last six months**?  Response range : 0 – 1000  **In the last six months, have you had sex with one or more casual male partners?**  Response: Yes; No  **In the last six months, have you penetrated or been penetrated by your casual male partner(s)?**  Response: Yes; No  **In the last 6 months, to avoid contracting HIV during AI with your casual male partner(s)...**  **b. Did you use condoms when you penetrated him/them?**  **a. Did he/they use condoms when he/they penetrated you?**  Response: Always; Often; Occasionally; Never; Not concerned. One response per item |
| Has your patient/client used methamphetamine or inhaled nitrites (popper) in the prior 6 months? | **During your last AI, apart from alcohol, cannabis or poppers, did you use at least one psychoactive substance (cocaine,**  **GHB/GBL, amphetamines, MDPV, 3-MMC, 4-MMC)?**  Responses: Yes; No; Don't know | Drug use during sex (Chemsex) | **During your last anal intercourse, apart from alcohol, cannabis or poppers, did you use at least one psychoactive substance (cocaine,**  **GHB/GBL, amphetamines, MDPV, 3-MMC, 4-MMC)?**  Response: Yes; No; Don't know |
| Does your patient/client have gonorrhea, chlamydia or syphilis, or does he have a history of these infections? | **In the past 12 months, have you had at least one screening test for**...  Hepatitis C; Hepatitis B; Syphilis; Chlamydia; Gonorrhea; HPV.  Responses: Yes; No. One answer per item  **If yes**  **What was the diagnosis of this screening test?**  Responses: Negative (not infected);Positive (infected); Don't know | STI in previous 12 months | **In the past 12 months, have you had at least one screening test for each of the following:**.  Hepatitis C; Hepatitis B; Syphilis; Chlamydia; Gonorrhea; HPV  Response: Yes; No. One answer per item  **If yes**  **What was the diagnosis of this screening test?**  Response: Negative (not infected);Positive (infected); Don't know |

AI: anal intercourse; TasP: treatment as prevention; PrEP: pre-exposure prophylaxis; GHB: gamma-hydroxybutyrate; GBL: Gamma-butyrolactone; MDPV: Methylenedioxypyrovalerone; 3-MMC: 3-methylmethcathinone; 4-MMC: 4-methylmethcathinone; HPV: human papillomavirus;
